# Distinct Roles of SELENOF in Different Human Cancers

**DOI:** 10.3390/biom13030486

**Published:** 2023-03-06

**Authors:** Brenna Flowers, Oliwia Bochnacka, Allison Poles, Alan M. Diamond, Irida Kastrati

**Affiliations:** 1 Department of Cancer Biology, Loyola University Chicago, Maywood, IL 60153, USA; 2 Department of Pathology, College of Medicine, University of Illinois at Chicago, Chicago, IL 60612, USA

**Keywords:** cancer, selenoprotein F, SELENOF, redox protein quality control, energy metabolism

## Abstract

SELENOF, previously known as SEP15, is a selenoprotein that contains selenium in the form of the amino acid selenocysteine. Like other selenoproteins, the role for SELENOF in carcinogenesis has been investigated due to its altered expression compared to the corresponding normal tissue, its molecular function, and the association of genetic variations in the *SELENOF* gene to cancer risk or outcome. This review summarizes SELENOF’s discovery, structure, cellular localization, and expression. SELENOF belongs to a new family of thioredoxin-like proteins. Published data summarized here indicate a likely role for SELENOF in redox protein quality control, and in the regulation of lipids, glucose, and energy metabolism. Current evidence indicates that loss of SELENOF contributes to the development of prostate and breast cancer, while its loss may be protective against colon cancer. Additional investigation into SELENOF’s molecular mechanisms and its impact on cancer is warranted.

## 1. Introduction

Interest in the essential trace element, selenium, as a means to prevent the incidence and mortality from cancer was initiated in the 1970s when correlative studies indicated an inverse association between selenium status (both dietary intake and serum levels) and mortality from various cancers [1,2]. Human trials were complemented by animal studies demonstrating that higher dietary selenium levels could prevent chemically induced and spontaneous cancers in several tissues [3,4,5]. Selenium in a variety of forms may be of therapeutic value by inhibiting the transformed phenotype, by displaying increased toxicity to cancer cells, and by altering oncogenic signaling pathways (reviewed in [4,6,7,8,9]). These data led to the well-powered prostate cancer prevention study, the Selenium and Vitamin E in Cancer Prevention Trial (SELECT). In this North American trial supported by the National Institutes of Health, a 38,000 man cohort consisting of African Americans over the age of 50 or all other men over 55 were randomly divided into four groups, receiving either placebo, selenium, vitamin E or both selenium and vitamin E [10]. The study was terminated early, as there were indications that the vitamin E group experienced a higher risk of prostate cancer and there was no benefit from selenium supplementation [11]. However, the men in the selenium-supplemented group also experienced a greater risk of prostate cancer if their baseline levels were relatively high, although the greater risk seen in the vitamin E group was eliminated when the participants also received selenium [12]. In sum, epidemiological data still support an inverse association between selenium status and the risk for several cancers including prostate cancer; however, interventional trials have not demonstrated any prevention benefits to selenium supplementation [13].

The majority of early animal data on chemoprevention with selenium was focused on mammary carcinogenesis [3,4,5]. In the case of breast cancer, a review summarizing the most recent evidence did not support a preventive role of selenium on cancer incidence [14]. However, breast cancer patients in the lowest quartile of selenium status had a reduced cancer-specific survival compared to those in the highest quartile [14]. This indicates that selenium supplementation may be of therapeutic benefit in a subset of breast cancer patients with low selenium status. However, validation through intervention trials and a closer examination of patients’ baseline selenium levels and genetic differences are warranted.

Research on the potential role of selenium in cancer has shifted from searching for the benefits of selenium supplementation to assessing the impact of selenium containing proteins. These proteins fall into two classes: those with a covalently bound selenium atom, such as SELENBP1, and the larger group referred to as selenoproteins that contain selenium in the form of the amino acid selenocysteine. While the number of selenoprotein genes varies in the genomes of life forms throughout evolution, there are 25 known in the human genome [15,16,17,18]. The selenocysteine present in selenoproteins most often resides in the protein’s active site where it facilitates redox reactions at a much faster rate compared to cysteine [19]. This amino acid is inserted co-translationally in response to an in-frame UGA codon that would be recognized as a stop codon were it not for the presence of a stem and loop structure in the 3′-untranslated region (UTR) of the corresponding mRNA [20,21]. The process requires a specific selenocysteine tRNA that is aminoacylated with serine which is phosphorylated and subsequently converted to selenocysteine prior to being incorporated into the growing peptide [22,23,24].

The discovery of selenoproteins and their participation in antioxidant defense shifted the focus of research to individual selenoproteins and their potential role in cancer initiation and progression [22]. Some selenoproteins have been investigated in this regard due to altered levels in cancers compared to normal tissues, their molecular function, or the association of genetic variations in the corresponding genes to cancer risk or outcome [25,26]. For example, polymorphisms in the genes for members of the glutathione peroxidase family of antioxidant selenoproteins have been associated with the risk of several cancers, although epidemiological studies have often resulted in conflicting results [27,28]. Possible reasons for studies yielding different conclusions include the impact of selenium status or ethnicity of study participants [29,30]. One such protein, selenoprotein F (SELENOF), for which there is considerable evidence in cancer outcomes, is the focus of this review.

## 2. SELENOF’s Discovery, Structure, Cellular Localization and Expression

Initial efforts to identify selenium-containing proteins relied on the detection of radiolabeled proteins in the presence of ^75^Se followed by their physical separation. Using this approach, a strongly labeled 15 kDa protein was initially detected in rat prostatic tissue [31,32]. A ^75^Se-labeled protein of the same weight was detected and purified in a human T-cell line and the sequence of tryptic peptides led to the isolation of the corresponding cDNA [33]. The determined sequence of the cDNA included an in-frame TGA codon and the stem-loop structure in the 3′-UTR could function as a Selenocysteine Insertion Sequence (SECIS) element [34], thus verifying the protein as a selenoprotein. The gene for this 15 kDa selenoprotein protein was established to be 51 kb long and consisted of five exons and four introns. Sep15 was then renamed SELENOF to conform to an established nomenclature [35] and was found to be localized on chromosome 1p31, a genetic locus commonly mutated or deleted in cancer [34,36]. Subsequent analysis identified SELENOF homologues in the mouse, rat, C. elegans, and B. malayi, with SELENOF in the latter two species containing a cysteine instead of a selenocysteine [33].

The initial characterization of the 3′-UTR of SELENOF mRNA revealed two distinct stem-loop structures, with the latter one being able to efficiently support UGA recoding as determined using reporter constructs [33,34,37]. The SELENOF 3′-UTR is polymorphic with sequence variations at position 811 within the first stem loop described as the SECIS-like element and a second polymorphism at position 1125 in the SECIS element [34,37]. These polymorphisms are in disequilibrium and form a haplotype among all genomic sequences characterized to date. The haplotypes exhibit racial differences in frequency with the minor haplotype being 5–10 fold more abundant in the genomic DNA of cancers obtained from African Americans as compared to the DNA of cancers obtained from Caucasians [37,38].

Examining the levels of SELENOF mRNA using the available dbEST and CGAP databases suggests that the protein was most highly expressed in the thyroid and prostate [33,34]. In a panel of diverse human tissues, high levels of SELENOF, particularly in the prostate, were reported, but SELENOF was also observed in benign human breast, colon, and kidney tissue [38]. Pull-down experiments using extracts from mouse prostate and rat liver detected UDP-glucose:glycoprotein glucosyltransferase (UGGT) as a SELENOF binding partner and binding occurs through a cysteine-rich SELENOF domain [39,40]. This result was expanded upon by showing that SELENOF resides in the endoplasmic reticulum (EnR) and contains an N-terminal EnR localization signal but not a C-terminal EnR retention signal, and all of the SELENOF in these cells was bound to UGGT [39]. Except for benign prostate tissue where SELENOF localizes to the outer membrane, SELENOF staining appears diffused throughout the cytoplasm consistent with its EnR localization in other epithelial tissues such as breast and colon [38].

Considering that SELENOF is an EnR resident protein, its regulation by stress response pathways was investigated. In murine fibroblast cells, SELENOF was transcriptionally upregulated in response to adaptive EnR stress caused by tunicamycin and brefeldin A, whereas acute EnR stress caused by dithiothreitol and thapsigargin leads to rapid and specific degradation of SELENOF by proteasomes [41]. SELENOF levels are also responsive to selenium bioavailability. SELENOF mRNA expression in mouse liver and kidney was moderately regulated by dietary selenium compared to the highly sensitive glutathione peroxidase 1 (GPX1), SELENOH, or SELENOW [42]. Conversely, SELENOF levels assayed by Western blotting indicated that its expression was reduced in the liver and kidneys of mice maintained on a selenium-deficient diet as compared with those maintained with 0.1 and 0.4 ppm selenium [43]. Moreover, the minor SELENOF haplotype was less responsive to selenium with regard to SECIS-mediated UGA recoding efficiency [37]. One molecular mechanism of how stressful conditions or selenium availability regulate SELENOF gene expression implicates heat shock factor 1 [44]. *SELENOF* resides in the short arm of human chromosome 1 [34], a region frequently characterized by genetic alterations in cancers [36], predominantly deletions, indicating that loss of heterozygosity at the *SELENOF* locus may contribute to the reduction in SELENOF levels or the unmasking of deleterious recessive mutations. Other regulatory mechanisms remain largely unknown.

## 3. SELENOF’s Putative Roles in Redox Protein Quality Control and Metabolism

Using solution NMR spectroscopy, the structures of mouse SELENOM and the fruit fly ortholog of SELENOF were resolved and it was established that SELENOF and SELENOM are structural homologs that constitute a new thioredoxin-like protein family [43] (SELENOF PDB ID: 2A4H). SELENOF and SELENOM contain a central α/β domain that is composed of three α-helices and a mixed parallel/anti-parallel four-stranded β-sheet. SELENOF has an elongated cysteine-rich N-terminus which is highly conserved among its homologs but absent in sequence homologs of SELENOM. The location of the active-site redox motifs within the thioredoxin-like proteins fold together with the observed localized conformational changes after a thiol-disulfide exchange indicated that SELENOF and SELENOM have redox activity (see Figure 3 in [43]). The calculated redox potential (−225 mV) was intermediate between that of thioredoxin (−270 mV) and protein disulfide isomerase (−175 mV), suggesting that SELENOF may catalyze the reduction and/or isomerization of disulfide bonds by functioning as a weak reductant or protein disulfide isomerase. As mentioned above, the cysteine-rich domain of SELENOF has an established function; it mediates the formation of a high affinity 1:1 complex between SELENOF and UGGT, the folding sensor of the calnexin cycle [40]. Based on SELENOF’s redox activity, its localization within EnR and its association with UGGT a putative role for SELENOF on modulating EnR stress response was postulated.

**Figure 1:**
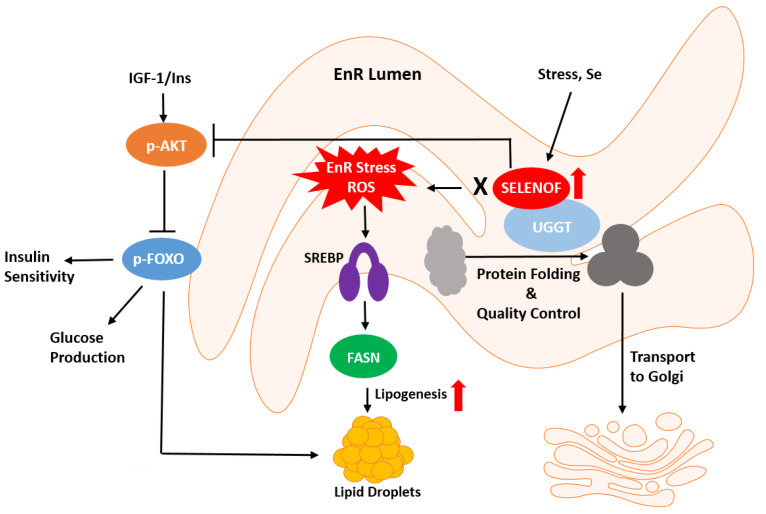
Schematic representation of SELENOF’s putative roles in redox protein quality control and metabolism. SELENOF in most tissues, except for normal prostate, resides in the endoplasmic reticulum (EnR). SELENOF associates with UGGT (UDP-glucose:glycoprotein glucosyltransferase) and regulates protein disulfide bond formation and redox quality control. SELENOF-dependent disruption of EnR homeostasis possibly through ROS (reactive oxygen species) leads to metabolic dysregulation, such as insulin insensitivity or glucose production via AKT (protein kinase B) and FOXO, (forkhead box O, a transcription factor), or lipogenesis via SREBP (sterol regulatory element binding protein, a transcription factor) and FASN (fatty acid synthase).

A role for SELENOF in the unfolded protein response (UPR), a molecular response to EnR stress induced by the accumulation of misfolded proteins, was investigated given that agents that induce EnR stress impacted the expression of SELENOF in murine fibroblast cells [41]. However, reducing the levels of SELENOF by RNA interference in these same cells did not result in increased EnR stress [41]. Instead, depletion of SELENOF in Chang liver cells, later determined to be HeLa cells, a cervical cancer line, produced a mild EnR stress response [45]. Using Selenof knockout (KO) cells and mice, a delayed transport of misfolded proteins from the EnR to the Golgi and elevated levels of misfolded immunoglobulins in the serum of the KO mice were reported [46]. This led the authors to suggest that SELENOF serves as a “gatekeeper” function controlling the secretion of glycoproteins [46]. This hypothesis is consistent with the observed development of cataracts in these mice which is likely due to the misfolding of proteins of the lens [47]. Alternatively, the reduction in SELENOF may induce oxidative stress in the lens as knocking down SELENOF in human lens epithelial cells enhanced tunicamycin-induced apoptosis without EnR stress being affected [48]. To summarize, the data thus far indicates a role for SELENOF in redox protein quality control in normal physiology but not as a master regulator of EnR stress response (Figure 1). For cancer cells to survive, they must resist numerous internal and environmental insults associated with neoplasia that jeopardize proteostasis within the EnR, therefore posing a different challenge for protein processing [49]. SELENOF’s impact on EnR proteostasis in the context of cancer remains unclear.

Recent data indicate that the depletion of SELENOF in cultured human cells or in Selenof KO animal models leads to differential expression of metabolism-related genes and associated phenotypes. Using immortalized human prostate epithelial cells, knockdown of SELENOF with an siRNA construct resulted in increased mitochondrial respiration and presumably ATP synthesis, increased phosphorylation and activation of AMP-activated protein kinase and inhibition by phosphorylation of acetyl CoA carboxylase, key proteins in regulating energy metabolism [50]. SELENOF was reported to bind to retinol dehydrogenase 11 and its overexpression led to reduced retinol production indicating that SELENOF may also play a role in vitamin A metabolism [51]. Proteomic analysis of liver tissue from Selenof KO male mice compared to wild-type C57BL/6J mice indicated 83 differentially expressed proteins involved in energy metabolism including fatty acid synthase, ATP-citrate synthase, and pyruvate kinase [52]. Furthermore, the levels of NADPH and ATP, the main energy currency of the cell, were significantly changed in the livers of Selenof KO mice. In a follow-up study, changes in lipoprotein lipase and carboxylesterase 1D, two lipid metabolism proteins, were reported indicating a broader metabolic impact [53]. Additional characterization of the Selenof KO mice did not reveal significant changes in glucose and insulin levels, although there was an increase in serum lipids. When provided with a high fat diet, fasting glucose and insulin levels were higher in the KO mice and the weights of the mice were significantly higher only in the KO mice with high fat diet. Overall, these data indicate that the loss of Selenof exacerbates the metabolic syndrome when animals are exposed to a high fat diet. In contrast, a more recent examination showed that glucose metabolism differences are significant in younger SELENOF KO mice (12 weeks old) via the disruption of redox homeostasis; however, these differences tended to dissipate with age (16 weeks and older) [54]. Metabolic dysregulation also occurs when diets with excess selenium are consumed by mice and rats [55,56]. In yellow catfish, excessive selenium intake (14mg/kg) increased SELENOF protein expression and glycogenolysis through the SELENOF-dependent AKT1-FOXO3a-PYGL axis, resulting in an increased lipid deposition and lipogenic enzyme activity [57]. In a similarly SELENOF-dependent manner, excessive selenium increased triacylglyceride and glucose contents, but reduced hepatic glycogen content. These results indicate a diet consisting of excess selenium impacts biological pathways such as glucose and lipid metabolism. Collectively, these data implicate a possible role for SELENOF in the regulation of lipids, glucose, and energy metabolism (Figure 1).

## 4. SELENOF Activities in Different Types of Cancers

SELENOF expression was initially reported to be high in the prostate compared to other tissues [33]. Additional interest in SELENOF in relation to prostate cancer was sparked by the observation that polymorphisms in *SELENOF* gene were associated with prostate cancer mortality among participants of the Physician’s Health Study [58]. Examination of human prostate tissue from two different tissue microarrays indicated that SELENOF co-localized with the plasma membrane in benign prostate tissue and immortalized human prostate cells in contrast to its location in other tissues where it resides in the EnR [38,50]. Moreover, SELENOF levels were significantly lower in prostate cancer samples compared to adjacent benign tissue, raising the possibility that the loss of SELENOF in prostate cancer was contributing to oncogenic progression. Support for this was obtained from cell culture studies where it was established that reducing SELENOF in RWPE-1 immortalized prostate epithelial cells resulted in the acquisition of the ability to grow in soft agar and enhanced migration as determined with the scratch assay [50]. Most cancer cells shift their energy needs to aerobic glycolysis with an increased rate of glucose uptake and lactate production, referred to as the Warburg effect [59]. However, normal prostate cells rely heavily on glycolysis to divert the metabolic intermediates for the production of citrate that is required to maintain sperm health [60]. Attenuating the production of SELENOF in RWPE-1 cells resulted in a shift in decreased oxygen consumption and phosphorylation of regulatory proteins that promote glycolysis, thus being more typical of prostate cancer cells [50]. A review of the effects of altering SELENOF levels on energy metabolism in prostate cells has recently been published [61].

SELENOF has been implicated in breast cancer based on the observation that loss of heterozygosity at the *SELENOF* locus occurs in breast tumors [37]. The *SELENOF* gene was mapped on chromosome 1 at the 1p31 region, which is commonly deleted in breast tumors and is indicative of this region potentially harboring putative tumor suppressors [36]. Data supporting a tumor suppressor function for SELENOF in breast cancer have been recently published [62]. *SELENOF* mRNA is significantly lower in late-stage patient tumor samples compared to normal breast tissue and lower SELENOF levels predict poor patient outcome measured as overall survival, recurrence-free survival, or distant metastasis-free survival. Enhancing SELENOF expression in breast cancer cells with low endogenous levels of SELENOF elicited anti-cancer activities by reducing proliferation and significantly increasing cell death. Furthermore, SELENOF overexpression attenuates a number of aggressive cancer phenotypes in breast cancer cells, including clonogenic survival and mammosphere formation, and enhances the response to drugs or radiation used in breast cancer therapy. Consistent with in vitro observations, SELENOF overexpression also attenuated xenograft tumor growth. Conversely, silencing SELENOF in breast cancer cells with relatively high levels of SELENOF led to an increase in Ki67, a proliferation marker, and stemness genes. When the mouse mammary glands from Selenof KO mice were compared to age-matched wild-type mice, it showed a significant increase in Ki67 and a reduction in p21, the cyclin-dependent kinase inhibitor, consistent with the SELENOF silencing data in human breast cancer cell lines and opposite to the SELENOF induction data.

The effect of SELENOF polymorphisms and loss of heterozygosity at position 1p31 has also been investigated in the context of malignant mesothelioma (MM), a cancer of the mesodermal layer most often associated with the lung [63]. After comparing 23 malignant mesothelioma cell lines to normal mesothelial cells, loss of heterozygosity was found to occur in the DNA of 20 of these lines, 12 of which had lower SELENOF expression. Selenium supplementation was most effective at inhibiting growth in cell lines homozygous for the 1125G allele, indicating this polymorphism may impact the effectiveness of the anti-proliferative properties of selenium. Furthermore, cell death from selenium supplementation was dependent on SELENOF. However, no differences in the polymorphism frequency were detected in patient samples versus normal tissues from MM patients or the normal population.

Results contrasting to those obtained with prostate and breast cancer models regarding the benefits of SELENOF in colon cancer models have been reported. SELENOF has the highest expression level out of the 24 selenoproteins expressed in mice in the murine colorectal cancer cell line, CT26 [64]. Reducing SELENOF levels in these cells using shRNA resulted in reduced cell growth and the ability of these cells to grow in soft agar. Furthermore, mice injected with SELENOF knockdown CT26 cells developed more tumors and pulmonary metastasis compared to mice injected with control cells. Genes related to cell cycle, such as cyclin B1, were differentially regulated in between wild-type and SELENOF knockdown cells, and SELENOF knockdown cells showed a higher percentage of cells in G2/M. Similarly, SELENOF knockdown in the HCT116 and HT29 human colorectal cancer cell lines resulted in decreased ability to grow in soft agar and smaller colonies [65]. SELENOF knockdown cells also had reduced proliferation as determined by FACS analysis, which showed an increased percentage of cells in G0/G1 phase [65]. In an in vivo model using Selenof KO, wild-type and heterozygous mice, azoxymethane-induced aberrant crypt formation (a precancerous lesion of the colon) was evaluated [66]. Selenof KO mice had fewer aberrant crypts than heterozygous and wild-type mice, even when fed a selenium-supplemented diet of 0.1 μg selenium/g. A microarray analysis of the colon showed that in Selenof KO mice, guanylate binding protein 1 (GBP-1) was increased 20-fold while pathway analysis showed that “cellular development, growth, and proliferation” was the top altered pathway between Selenof KO and WT mice [66]. Another study evaluated how loss of SELENOF impacted the azoxymethane- and dextran sulfate sodium-induced inflammatory colon carcinogenesis [67]. There was no significant difference in the number of tumors developed between Selenof KO mice and their wild-type littermates, despite Selenof KO mice having fewer aberrant crypt formations. It was argued that this may be due to differences in intestinal structure and barrier integrity caused by the loss of Selenof. More importantly, this highlights differences emerging based on the carcinogenesis model used. To summarize, these studies using murine and human colorectal cell lines, together with the Selenof KO mouse model, indicate that loss of SELENOF is protective in the context of colorectal cancer and the formation of precancerous lesions [64,65,66]. Table 1 summarizes the cancer studies discussed above. 

## 5. Conclusions

As described above, loss of SELENOF in human breast or prostate cell lines resulted in the acquisition of a transformed or aggressive cancer phenotype [50,62]. In contrast, loss of SELENOF in mice or human colon cancer cells resulted in attenuation of the same phenotypes [64,65] and SELENOF-deficient mice were more protected from azoxymethane-induced aberrant crypt formation [66,67]. The reasons for these apparently opposite results in the different tissue types cannot be explained at this time. In normal prostate cells, SELENOF co-localizes with the outer cell membrane while in other tissues, including those of the breast and colon, SELENOF is predominantly an EnR-associated protein [38,41]. Despite SELENOF’s different subcellular localizations in breast and prostate tissues, there are commonalities between malignancies that arise in these organs. Breast and prostate cancers are the two most common invasive cancers in women and men, respectively [68]. Although these cancers arise in organs that are different in terms of anatomy and physiological function, both organs require gonadal steroids for their development, and tumors that arise from them are typically hormone dependent and have remarkable underlying biological similarities [69]. Epidemiological studies on women over the past decades have consistently shown that an increase in female hormones, such as estrogens and progestin as a result of pregnancy or use of exogenous steroid hormones, is associated with a lower risk for developing colorectal cancer [70]. Less is known about the association between sex hormone levels and colorectal cancer risk in men.

There are several aspects of the immunology associated with breast and colorectal cancer that are distinct. For example, the presence of tissue regulatory T cells is associated with worse prognosis in breast cancer [71] but improved prognosis in colon cancer [72]. Similarly, tumor-associated macrophages (TAMs), a major innate immune cell type of human tumors, are tumor promoting in breast cancer but protective in colorectal cancers [73]. In this regard, SELENOF knockdown in mouse colorectal carcinoma CT26 cells resulted in changes in the expression of inflammatory-related pathways, particularly in pathways regulated by interferon [74].

The data obtained using in vitro and in vivo cancer models should be interpreted cautiously by considering the model used, hormone status, and immune components present. Moreover, the impact of SELENOF on tumor initiation or promotion may be affected by the means of cancer induction (chemical, genetic or spontaneous) and tissue type. A better understanding of the role of SELENOF in cancer risk and development will also require a greater understanding of SELENOF’s function in cells within various tissue types.

## Figures and Tables

**Table 1:** A summary table of the impact on SELENOF in various cancers is included.

Cancer	Model	Phenotype	Reference(s)
**Prostate Cancer**	Microarray Serum selenium levels Genotyping SELENOF knockdown	↓SELENOF correlates to *increased* cancer progression	[38,50,60]
**Breast Cancer**	The Cancer Genome Atlas data SELENOF overexpression SELENOF knockdown Selenof KO mice	↓SELENOF correlates to *increased* proliferation and *decreased* cell death	[62]
**Colorectal Cancer**	SELENOF knockdown Selenof KO mice	↓SELENOF correlates to *decreased* proliferation and aberrant crypt formation	[64,65,66,67]
**Malignant Mesothelioma**	SELENOF knockdown Selenium supplementation Genotyping Patient samples	Effectiveness of selenium supplementation dependent on polymorphism	[63]

Arrow: Indicates reduced levels.

## Data Availability

Not applicable.
